# Estrogen and Visceral Nociception at the Level of Primary Sensory Neurons

**DOI:** 10.1155/2012/960780

**Published:** 2011-08-23

**Authors:** Victor Chaban

**Affiliations:** ^1^Charles R. Drew University of Medicine and Science, Los Angeles, CA 90095, USA; ^2^David Geffen School of Medicine, University of California, Los Angeles, Los Angeles, CA 90095, USA

## Abstract

Clinical studies suggest the comorbidity of functional pain syndromes such as irritable bowel syndrome, painful bladder syndrome, chronic pelvic pain, and somatoform disorders approaches 40% to 60%. The incidence of episodic or persistent visceral pain associated with these “functional” disorders is two to three times higher in women than in men. One of the possible explanations for this phenomenon is estrogen modulation of viscerovisceral cross-sensitization. While a central site of this modulation has been shown previously, our studies suggest a peripheral site, the dorsal root ganglion (DRG). Estrogens have remarkably wide range of functions including modulation of voltage-gated calcium channels (VGCCs) and purinoreceptors (P2Xs). Significantly, inflammation dramatically alters purinoception by causing a several fold increase in ATP-activated current, alters the voltage dependence of P2X receptors, and enhances the expression of P2X receptors increasing neuronal hypersensitivity. Gonadal hormones are thought as indispensable cornerstones of the normal development and function, but it appears that no body region, no neuronal circuit, and virtually no cell is unaffected by them. Thus, increasing awareness toward estrogens appears to be obligatory.

## 1. DRG Neurons and Visceral Sensitization

Sex hormones and 17*β*-estradiol (E_2_), in particular, directly influence the functions of primary afferent neurons. However, E_2_ has a multiplicity of actions: membrane, cytoplasmic, and nuclear: E_2_ modulates cellular activity by altering ion channel opening, G-protein signaling, and activation of trophic factor-like signal transduction pathways [1]. DRG neurons in culture express receptors of nociceptive signals [2] and retain most, if not all, their intracellular signaling cascades. DRG neurons *in vitro *are a valuable preparation because adult primary sensory neurons can be studied without the interference of modulation by central or peripheral messengers. Visceral afferents are sensitive to ATP [3], and several indirect pieces of evidence suggest that visceral afferents are E_2_-sensitive: (i) visceral pain is affected by hormonal level in cycling females [4]; (ii) there are gender differences in the prevalence of functional disorders involving the viscera [5]; (iii) putative visceral afferents [6] fit into the population of DRG neurons that are E_2_-sensitive. Although it is generally accepted that each primary afferent neuron is a single sensory channel, several studies have challenged that view and demonstrate that a population of DRG neuron can innervate both the viscera and somatic tissues. 

Both subtypes of estrogen receptors (ER*α* and ER*β*) are present in small-diameter DRG neurons, presumably nociceptors. Relevantly, estrogen receptors are distributed in regions of the central and peripheral nervous system that mediate nociception. A large body of the literature supports the idea that E_2_ modulates nociceptive responses in pelvic pain syndromes; however, whether E_2_ is pro- or antinociceptive remains unresolved. Within the context of our hypothesis, E_2_ modulation of nociceptive response depends on the type of pain, its durations, and the involvement of other antinociceptive mechanisms. Visceral nociception and nociceptor sensitization appear to be regulated by ATP and substance P [7], and DRG is an important site of visceral afferent convergence and cross-sensitization.

**Figure 1:**
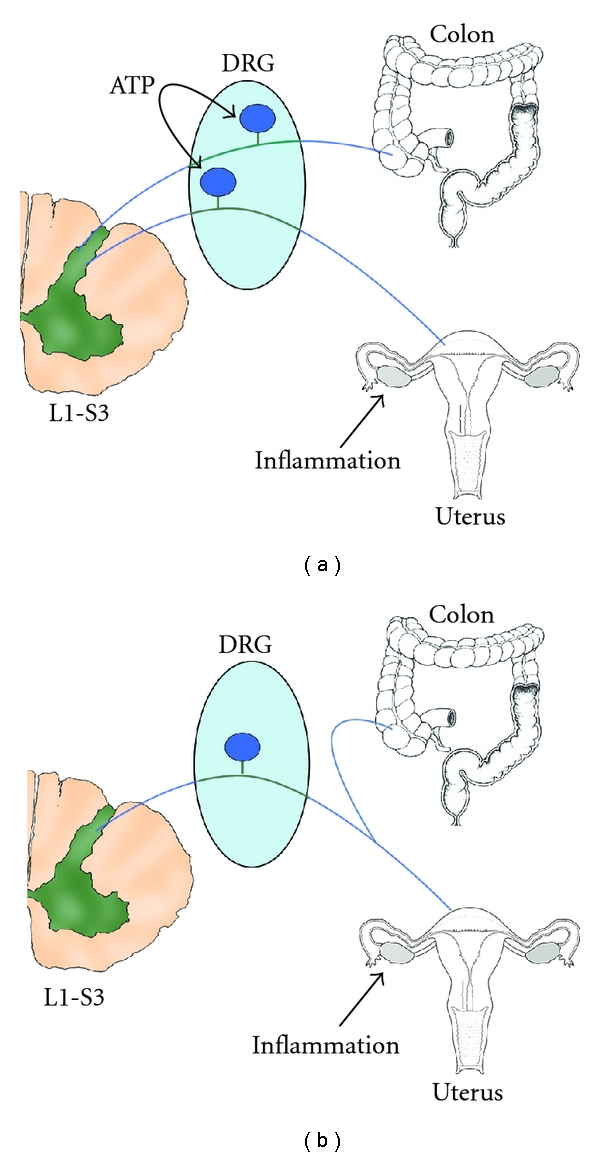
Models of alternative possibilities for viscero-visceral cross-sensitization in the DRG neuron. (a) ATP released by a neuron innervating the inflamed uterus acts on a neighboring neuron sensitizing its responses to colonic distention. (b) The same neuron innervates the uterus and colon. Uterus inflammation directly sensitizes the neuron to colonic distention.

Mechanisms of viscerovisceral hyperalgesia between organs with documented partially common sensory projection probably involve sensitization of visceroviscero-somatic convergent neurons [8]. Within the context of our hypothesis, sensory neurons can release an ATP intraganglionically during inflammation. The inflammation in the reproductive tract can cross-sensitize the response to ATP in colonic DRGs. Although it has been accepted that each primary afferent neuron is a single sensory channel (Figure 1(a)), several studies has challenged that view [9], and our own data using retrograde labeling demonstrate that population of DRG receive sensory input from different visceral organs: uterus and colon [10] and that inflammation in the uterus upregulated nociceptive signaling in the colon [11] (Figure 1(b)).

## 2. Transduction of Visceral Nociception

Pain is an unpleasant sensory and emotional experience associated with actual or potential damage or depicted in terms of such damage and normal transmitting pain system is absolutely essential to keep the integrity of our body [12]. The incidence of persistent, episodic, or chronic “functional” visceral pain associated with functional disorders such as irritable bowel syndrome, fibromyalgia, painful bladder syndrome, chronic pelvic pain, and others is much more prevalent in females [13, 14]. A meta-analysis of acute experimental pain studies (pressure pain and electrical stimulation) using a “box score” methodology in humans shows that females display greater sensitivity and that pain threshold and tolerance are modulated across the menstrual cycle [4]. 17*β*-estradiol (E_2_) may account for these observed fluctuations in pain perception and symptoms [13]. Thus, a possible explanation is that E_2_  may have different actions on nociceptive signaling depending on the origin of the noxious stimulus. The localization of both estrogen receptors ER*α* and ER*β* in DRG neurons and the attenuation of ATP-induced [Ca^2+^]_i_ [15] strongly suggest that E_2_ modulates visceral pain processing peripherally. 

Nociceptors were defined as receptors by Sherrington more than a century ago (1906) that respond selectively to stimuli that cause tissue damage. The nociceptors can be divided into two main groups based on anatomical and functional properties: Activity in myelinated A*δ*-fiber nociceptors gives rise to the initial sharp feeling of pain while on the contrary unmyelinated C-fiber nociceptors gives rise to the later, duller, burning pain sensation due to their different morphology. The cell bodies of these axons are located in the DRG.

The molecular and cellular mechanism of nociception has been defined during the past two decades. Molecular function of nociceptors depends on expression of different nociceptive receptors and ion channels that can be activated by noxious stimuli. These receptors and ion channels detect specific physical or chemical stimuli and induce membrane depolarization. Among these nociceptive receptors and ion channels, transient receptor potential ion channel (TRP)-related TRPV family and purinergic receptors (P2X_2_ and P2X_3_) have been very well studied and acknowledged as molecular detectors of noxious thermal, mechanical, or chemical stimuli [16]. Visceral nociceptive C-fibers are activated by ATP released by noxious stimuli from cells in target organs and have been implicated as mediators of noxious stimulus intensities [3]. Alteration in signal transduction of primary afferent neurons can result in enhanced perception of the visceral sensation, which is common in patients with different disorders resulting in elevated pain perception. Peripheral sensitization can develop in response to sustain stimulation, inflammation, and nerve injury.

## 3. Nongenomic Mechanism of Estrogen Action on Visceral Nociceptors

Both estrogen receptor *α* (ER*α*) and estrogen receptor *β* (ER*β*) belong to nuclear receptors of the steroid/thyroid superfamily, in which the members have structural and functional similarities such as a ligand-dependent transcription factor that modulates gene expression. There is a high homology between ER*α* and ER*β* in the DNA-binding domain (97%), but a moderate homology in the ligand-binding domain (55%), resulting in somewhat lower affinities for endogenous 17*α*- and 17*β*-estradiol to ER*β* than ER*α*. 

A variety of cell types respond rapidly to E_2_, making a nongenomic mechanism of action. Some nongenomic activities of estrogens may be explained by the presence of classic estrogen receptors [17–19], but a brain-specific ERX has also been suggested [20]. However, this putative new receptor has been expressed only in glial cells and can be activated by the inactive 17*α*-estradiol stereoisomer. Overall, the nongenomic estrogen effects are thought to be mediated by ER*α* that is sequestered to the cytoplasm or the plasma membrane within a signaling complex [1]. The initial evidence for this concept was provided by studies showing that signal transduction is initiated by estradiol conjugated to large, membrane impermeant molecules such as bovine serum albumin [15]. The local synthesis of estrogens in the brain provides high concentrations of estrogens locally. In fact, local synthesis of estrogens in the brain seems to be a requirement for the rapid signaling of estrogen receptor since the cyclic changes of plasma hormone levels are too slow to fit this rapid pattern of activation. Many effects of estradiol in the brain, such as part of the regulation of lordosis behavior [21] and signaling in the dorsal root ganglion [7, 18], are in the time range proposing nonclassic mechanisms.

ERs distributed through CNS and PNS including regions that mediate nociception. For example, ERs are expressed in dorsal horn neurons of the spinal cord [22, 23] and DRG neurons [24]. DRG neurons express both ER*α* and ER*β* *in vivo* [25] and *in vitro* [10]. These findings suggest that E_2_ may modulate sensory input at the primary afferent level. E_2_ can alter gene transcription, resulting in pronociceptive (reducing *β*-endorphin expression) or antinociceptive (increasing enkephalin expression) changes of endogenous opioid peptides and opioid receptors [22, 26, 27]. E_2_ can modulate cellular activity by altering ion channel opening and second messenger signaling by stimulating G-proteins [15, 28–30], the signal transduction pathways traditionally associated with membrane receptor activation. Many of these effects have been ascribed to membrane-associated receptors [31]. The results from other laboratories [28, 32] and our data [15] indicate that E_2_ is acting to modulate L-type VGCC. 

Adenosine 5′-triphosphate (ATP) is one of the most common chemical compounds in living cells. Neurons represent a remarkable source of ATP, as a neurotransmitter, which is widely used in both central and peripheral nervous systems [33]. ATP can be released from vesicle pools, or it can be coreleased and/or costored together with classical neurotransmitters in neurons. A decade ago, ATP was shown to be an extracellular signal involved in peripheral hyperalgesia [34]. One of ATP's best defined roles has been described in sensory transduction of noxious stimuli by activating purinergic, ATP-gated P2X receptors on primary afferent fibers [33]. ATP release from tissues during pathological conditions that cause tissue damage or inflammation activates P2X receptors on primary afferent fibers innervating the afferent organs [35]. 

Some molecular targets have been identified as key players in the activation and sensitization of visceral nociceptors, notably ASICs, TTX-resistant Na channels, and the TRPV1 receptor [36]. TRPV1 receptor is a sensory neuron-specific cation channel which belongs to the transient receptor potential subfamily 1 and plays an important role in transporting thermal and inflammatory pain signals. Evidence for TRPV1's role in their pathogenesis comes from studies showing that mice lacking TRP1R gene have deficits in thermal- or inflammatory-induced hyperalgesia [37]. Activation of both TRPV1 and P2X receptors induce mobilization of [Ca^2+^]_i_ in cultured DRG neurons [38]. Capsaicin-induced TPRV1 receptor-mediated changes in [Ca^2+^]_i_ may represent a level of DRG activation to noxious cutaneous stimulation, while ATP-induced changes in [Ca^2+^]_i_ may reflect the level of DRG neuron sensitization to noxious visceral stimuli since ATP is released by noxious stimuli and tissue damage near the primary afferent nerve terminals [39].

The expression of transient receptor potential vanilloid 1 (TRPV1) receptors is widespread in several areas of the nervous system, but it is particularly strong in DRG. TRPV1 receptors are at the cellular membrane as well as in the membrane of intracellular calcium stores (e.g., endoplasmatic reticulum). For pain transmission, the pain/temperature sensitive TRPV1 receptor is highly expressed in nociceptive neurons of the peripheral nerve system. TRPV1 receptors are activated by a wide variety of stimuli, both exogenous (capsaicin, noxious heat) and endogenous (protons, lipoxygenase products, anandamide, dopamine), and they mediate a nonselective cationic current with particularly high Ca^2+^ permeability [37]. The potentiating of TRPV1 activity can be quantified by measuring the differences of capsaicin-induced Ca^2+^ concentration changes before and after receptor activation [40]. Significantly, a subset of DRG neurons respond to both capsaicin and ATP [7] indicating that there may be cross-activation of these receptors that may underlie the sensitization of visceral nociceptors.

**Figure 2:**
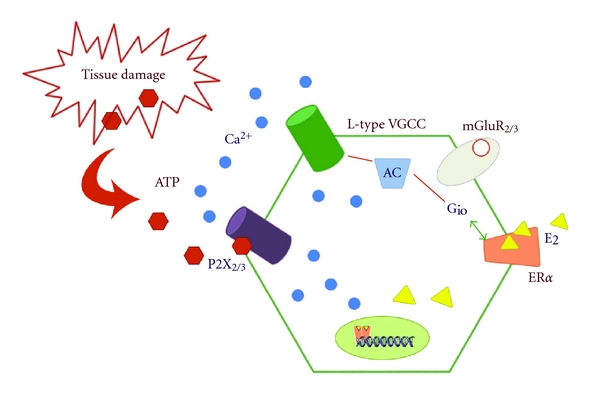
Proposed mechanism of estradiol effect on ATP-induced [Ca^2+^]_i_ signaling in primary sensory neurons. ATP released by tissue damage acts on P2X_3_ receptor resulting in activation of the L-type voltage-gated calcium channel (VGCC). ER*α* activates mGluR2/3 which in turn activates Gi/o signaling resulting in inhibition of adenylate-cyclase (AC). Decreased cAMP concentration reduced PKA activation and decreased the conductance of the L-type VGCC.

The action of estrogens at the level of primary sensory neurons is complex and determined by the characteristics of the target genes and coregulators, as well as, the regional availability of E_2_. Several authors have suggested that the sex differences in pain sensitivity and the prevalence of chronic pain disorders may result from a malfunctioning endogenous pain inhibitory response rather than an increase nociceptive activity [41]. Many previous studies have established connections between estrogens and the modulation of different nociceptive pathways. One of the mechanisms may be estrogen inhibition of nociceptive signaling (mediated by both P2X3 and TRPV1) through interaction with mGluR_2/3_. Painful stimuli initiate action potentials in the peripheral terminals of DRG neurons evoking release of excitatory neurotransmitters such as glutamate (Figure 2). Ligand-gated P2X receptors which can be activated by endogenous ATP during induction of action potentials are highly expressed in identified nociceptors [42]. Thus, E_2_-modulated encoding of nociceptive stimuli at the level of primary sensory neurons may contribute to our understanding of complex mechanism of nongenomic effect of gonadal steroids.

## 4. Conclusions

The fact that homeostatic changes are similar in all “functional” disorders suggests a model in which alteration in the neuronal circuits in predisposed individuals is triggered by the similar pathophysiology. Both physiologic and psychological variables appear to play important roles in the development of functional syndromes, and psychologically oriented treatments have a role in their management. Patients have an altered perception of visceral sensation that is typically manifested as hyperalgesia, an enhanced perception of pain. Pain is the symptom that patients with functional disorders list as the most depressing and is a major factor for consulting a physician. The functional characteristics of primary sensory (small diameter, presumably nociceptors) neurons and their receptors are usually investigated by means of a wide diversity of experimental models, both *in vivo *and *in vitro*. Several *in vivo *animal models have been developed and characterized for specific sensory modalities, including nociception, particularly in rodents [2]. The use of these models suggests the advantage of a physiological/pathological condition, and it is hence a basic step in the understanding of the fundamental mechanisms underlying the function of sensory systems and for the development and proof of new possible strategies for their modulation. As far as the modulation of receptors involved in nociceptive input transmission is concerned, it is indeed possible to reproduce in rodents various painful states observed in humans. The deficiency of suitable animal models for certain pain syndromes requires the use of strictly controlled *in vitro *paradigms in order to dissect the fundamental properties of the basic processes. One possible approach to the study of the functional characteristics of neurotransmitter (i.e. ATP) release and activated ion channel receptor is the direct recording of the receptor response to its agonist and antagonist on isolated cells expressing the receptor, by means of calcium imaging or patch clamp recordings techniques. Histological and functional studies implied that the important receptors involved in the transmission and modulation of nociceptive inputs are expressed at the level of the peripheral and/or central terminals of DRG neurons (e.g., P2X_3_ and TRPV1).

Although it is generally accepted that each primary afferent neuron is a single sensory channel, several studies have challenged that view and demonstrate that a population of DRG neuron can innervate both the viscera and somatic tissues. The design of the proposed studies using retrograde labeling from the uterus and colon will address the possibility that the same primary afferent can innervate both reproductive and gastrointestinal organs (DRG neuron will have both retrograde tract tracing dyes). This new subset of dichotomizing fibers provides a novel pathway for sensitization of one viscus by another. Adult DRG neurons in short-term culture retain the expression of receptors (P2X and TRPV1) [2] which mediate the response to putative nociceptive signals. They continue to respond to ER agonists mimicking *in vivo *activation. An important advantage is that these neurons can be studied apart from endogenous signals. A large body of literature supports the idea that E_2_ modulates nociceptive responses in functional pain syndromes. Within the context of our hypothesis, E_2_ modulation of nociceptive responses depends on the type of pain, its durations, and the involvement of other antinociceptive mechanisms. E_2_ modulates DRG neurons response to ATP, suggesting that visceral afferent nociceptors are modulated by E_2_, which may explain the observed clinical and animal gender differences in visceral hypersensitivity and suggests a potential target for mediating nociception. Future direction of research should include more experiments to study the role of peripheral ERs in visceral nociception using *in vivo *models. Thus, from a public health perspective, the outcome of this study will have a substantial impact, because it will increase our knowledge of nociceptive functional diseases, such as IBS, interstitial cystitis, and chronic pelvic pain, and help achieve a deeper understanding of gender differences presented in clinical aspects of these symptoms associated with various psychiatric disorders. Only a thorough understanding of the mechanism implicated in these phenomena can truly contribute to the design of new and more efficient therapies. Many illnesses affect women and men differently. Some disorders are more common in women, and some express themselves with different symptoms. It is very well documented that differences between the sexes exist in the prevalence and severity of a broad range of diseases, disorders, and conditions. In calling for greater focus on gender-based biomedical research, the clinical and scientific communities will be better equipped to identify barriers to the achievement of knowledge about gender differences-including ethical, financial, sociological, and scientific factors. In developing countries, pain accounts for nearly 20% of all primary health care visits. Studies have shown that at least one-third of patients with pain also suffer from depression and it affects more women than men. The new approach in pain research and treatment should address a crucial question in women's health and in visceral nociception in particular. Reaching further, this research is a liaison between the basic science work and the clinical aspects that are addressed through other disciplines such as anesthesiology (pain management), gastroenterology, obstetrics, and gynecology.
